# Anti-Neurofascin 155 Antibody-Positive Chronic Inflammatory Demyelinating Polyneuropathy/Combined Central and Peripheral Demyelination: Strategies for Diagnosis and Treatment Based on the Disease Mechanism

**DOI:** 10.3389/fneur.2021.665136

**Published:** 2021-06-10

**Authors:** Jun-ichi Kira

**Affiliations:** ^1^Translational Neuroscience Center, Graduate School of Medicine, and School of Pharmacy at Fukuoka, International University of Health and Welfare, Fukuoka, Japan; ^2^Department of Neurology, Brain and Nerve Center, Fukuoka Central Hospital, International University of Health and Welfare, Fukuoka, Japan

**Keywords:** chronic inflammatory demyelinating polyneuropathy, combined central and peripheral demyelination, neurofascin 155, node of Ranvier, IgG4

## Abstract

Chronic inflammatory demyelinating polyneuropathy (CIDP) is an immune-mediated demyelinating disease of the peripheral nervous system (PNS). A small number of CIDP patients harbors autoantibodies against nodal/paranodal proteins, such as neurofascin 155 (NF155), contactin 1, and contactin-associated protein 1. In most cases, the predominant immunoglobulin (IgG) subclass is IgG4. Node/paranode antibody-positive CIDP demonstrates distinct features compared with antibody-negative CIDP, including a poor response to intravenous immunoglobulin. The neuropathology of biopsied sural nerve shows Schwann cell terminal loop detachment from axons without macrophage infiltration or inflammation. This is partly attributable to IgG4, which blocks protein–protein interactions without inducing inflammation. Anti-NF155 antibody-positive (NF155^+^) CIDP is unique because of the high frequency of subclinical demyelinating lesions in the central nervous system (CNS). This is probably because NF155 coexists in the PNS and CNS. Such cases showing demyelinating lesions in both the CNS and PNS are now termed combined central and peripheral demyelination (CCPD). NF155^+^ CIDP/CCPD commonly presents hypertrophy of spinal nerve roots and cranial nerves, such as trigeminal and oculomotor nerves, and extremely high levels of cerebrospinal fluid (CSF) protein, which indicates nerve root inflammation. In the CSF, the CXCL8/IL8, IL13, TNFα, CCL11/eotaxin, CCL2/MCP1, and IFNγ levels are significantly higher and the IL1β, IL1ra, and GCSF levels are significantly lower in NF155^+^ CIDP than in non-inflammatory neurological diseases. Even compared with anti-NF155 antibody-negative (NF155^−^) CIDP, the CXCL8/IL8 and IL13 levels are significantly higher and the IL1β and IL1ra levels are significantly lower than those in NF155^+^ CIDP. Canonical discriminant analysis revealed NF155^+^ and NF155^−^ CIDP to be separable with IL4, IL10, and IL13, the three most significant discriminators, all of which are required for IgG4 class switching. Therefore, upregulation of both Th2 and Th1 cytokines and downregulation of macrophage-related cytokines are characteristic of NF155^+^ CIDP, which explains spinal root inflammation and the lack of macrophage infiltration in the sural nerves. All Japanese patients with NF155^+^ CIDP/CCPD have one of two specific human leukocyte antigen (HLA) haplotypes, which results in a significantly higher prevalence of HLA-DRB1^*^15:01-DQB1^*^06:02 compared with healthy Japanese controls. This indicates an involvement of specific HLA class II molecules and relevant T cells in addition to IgG4 anti-NF155 antibodies in the mechanism underlying IgG4 NF155^+^ CIDP/CCPD.

## Introduction

**Figure 1:**
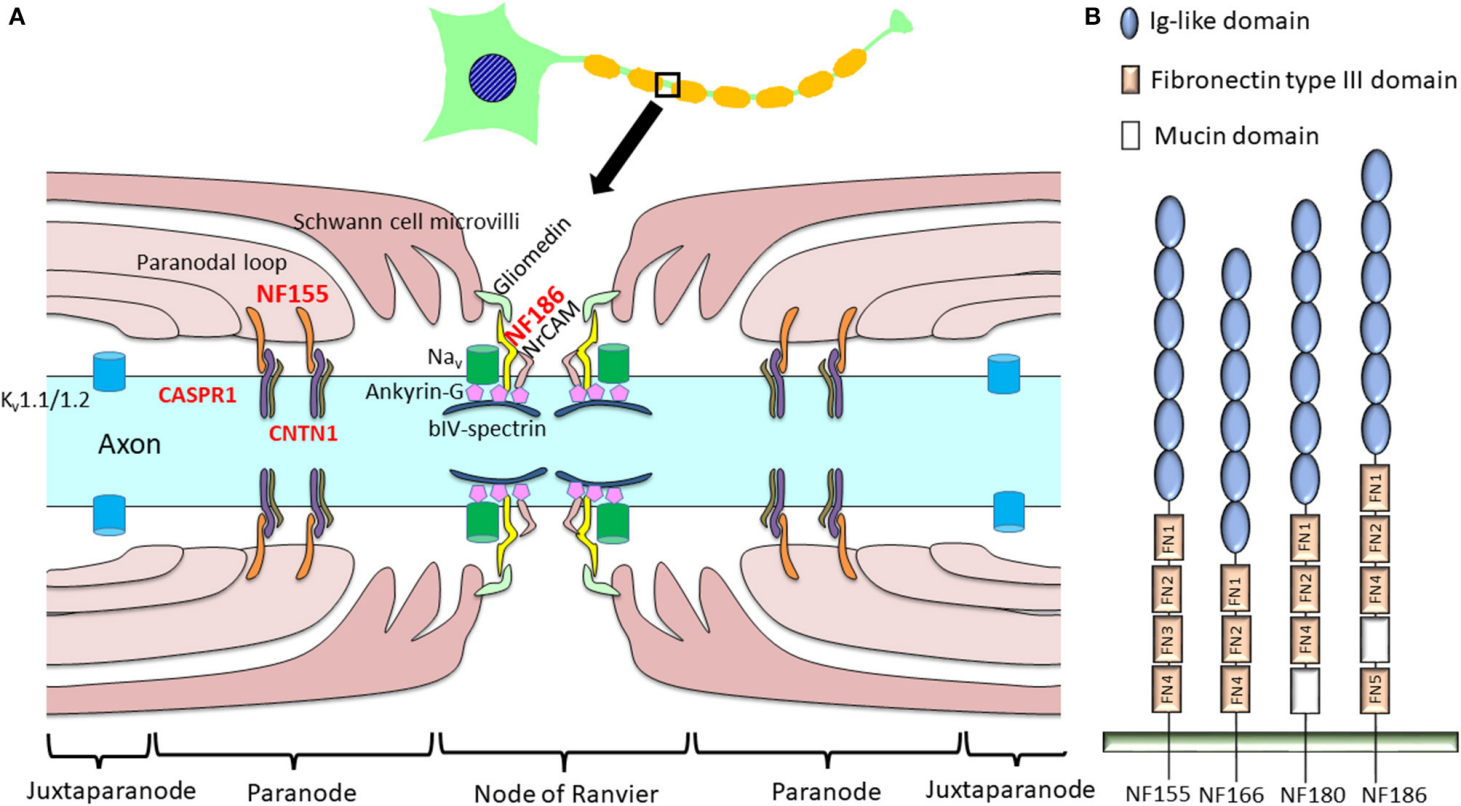
Schema of a node of Ranvier indicating proteinaceous antigens targeted by anti-nodal/paranodal antibodies in chronic inflammatory demyelinating polyneuropathy (CIDP). **(A)** In CIDP, autoantibodies to NF155, NF186, CNTN1, and CASPR1 have been discovered, while antibodies against NF186, gliomedin, and contactin were also reported in a minority of patients with Guillain–Barré syndrome (28). **(B)** Schematic diagram of the different neurofascin isoforms. In the mature nervous system, neurons express NF186 while oligodendroglia express NF155. NF180 and NF166 are present in immature neurons (17). NF155 and NF186 differ in their extracellular domains: NF155 has FN3, but NF186 lacks this domain and instead harbors a mucin domain between FN4 and FN5. CASPR1, contactin-associated protein 1; CNTN1, contactin 1; FN, fibronectin type III domain; Ig, immunoglobulin; K_v_, potassium channel; Na_v_, sodium channel; NF, neurofascin; NrCAM, neuronal cell adhesion molecule.

Chronic inflammatory demyelinating polyneuropathy (CIDP) is the most common acquired immune-mediated neuropathy that affects myelinated fibers. CIDP is etiologically heterogeneous, which results in variable responses to immunotherapies. Accumulating evidence indicates that a fraction of CIDP patients carries autoantibodies against nodal or paranodal proteins (Figure 1A), such as neurofascin (NF) 155 (NF155) (1–7), neurofascin 186 (NF186) (1), contactin 1 (CNTN1) (8–10), and contactin-associated protein 1 (CASPR1) (11). The individual autoantibodies are associated with unique features; therefore, CIDP associated with these nodal/paranodal autoantibodies is now recognized as autoimmune nodopathy or paranodopathy. In most cases, the predominant immunoglobulin (IgG) autoantibody subclass is IgG4 (1–11). Node/paranode antibody-positive CIDP presents distinct features compared with antibody-negative CIDP, including a poor response to high-dose intravenous immunoglobulin (IVIg) (1–11). This is, in part, attributable to the biological functions of IgG4, which does not elicit inflammation but blocks protein–protein interaction (12). Although overt central nervous system (CNS) manifestations are rare in CIDP, anti-NF155 antibody-positive (NF155^+^) CIDP frequently shows subclinical demyelinating lesions in the CNS, such as in optic nerves and cerebral white matter (3, 13). NF155 and other nodal antigens, such as CNTN1 and CASPR1, exist in both the peripheral nervous system (PNS) and CNS (14, 15). Thus, it remains to be elucidated why NF155^+^ CIDP involves the CNS more frequently compared with other nodal antibody-positive CIDPs. Cases showing demyelinating lesions in both the CNS and PNS are now termed combined central and peripheral demyelination (CCPD) (16). In this review, I describe the characteristic features of NF155^+^ CIDP/CCPD and strategies for diagnosis and treatment based on the underlying disease mechanism.

## Neurofascins

NF is crucial in constructing and maintaining the nodes of Ranvier. Four major NF polypeptides are produced by alternative splicing: NF186, NF180, NF166, and NF155 [Figure 1B; (17)]. These polypeptides are principally expressed in nervous tissues and comprise six immunoglobulin-like domains, up to five fibronectin type III (FN) domains, a transmembrane domain, and a short cytoplasmic domain. The mature nervous system predominantly expresses a neuronal isoform, NF186, and a glial isoform, NF155, whereas immature neurons express NF180 and NF166 (17). Extracellular domains are distinct between NF155 and NF186; NF155 harbors FN3, while NF186 lacks this domain and instead possesses a mucin domain between FN4 and FN5 (17).

Glial NF155 is expressed at paranodal loops of Schwann cells in the PNS (15) and in oligodendrocytes in the CNS (14). Glial NF155 acts as a cell adhesion molecule, interacting with axonal CNTN1 and CASPR1 (18). Together with axonal CNTN1 and CASPR1, glial NF155 forms septate-like transverse bands between the terminal loops and axons that play key roles in maintaining ion channel clustering at the nodes of Ranvier [Figure 1A; (15)]. Glia-specific deletion of NF155 produced a marked reduction of nerve conduction velocity together with the migration of paranodal CASPR1 and juxtaparanodal potassium channels (K_v_1.1) toward the nodal region (19). Therefore, NF155 is indispensable for the separation of nodal voltage-gated sodium channels (Na_v_) from juxtaparanodal potassium channels (K_v_). The loss of NF155 and CNTN1 in genetically engineered mice results in the disruption of septate-like junctions, which produces a large gap between the axolemma and Schwann cell terminal loops. This leads to a decreased nerve conduction velocity (15, 19, 20); therefore, these molecules are considered to be fundamental in maintaining saltatory conduction.

Axonal NF186 interacts with ankyrin-G to cluster Na_v_ at the nodal axolemma (21). Transgenic expression of NF155 in the myelinating glia in *Nfasc* knockout mice, which lack both NF155 and NF186, rescued Na_v_ and ankyrin-G clustering, while transgenic expression of NF186 in neurons alone also restored nodal Na_v_ and ankyrin-G clustering (22). Tissue-specific genetic ablation of NF186 in CNS and PNS neurons only induced loss of adhesion and extracellular matrix molecules, such as neuronal cell adhesion molecule (NrCAM) and gliomedin in the PNS and brevican in the CNS; however, Na_v_ still clustered at nodes, although this gradually decreased with aging (23, 24). Thus, NF186 is not essential but helps to maintain and stabilize Na_v_ at the nodes (25). Therefore, both NF155- and NF186-dependent mechanisms are important for node formation and maintenance (25).

Immature CNS and PNS tissues, NF166 and NF180 play critical roles in neurite outgrowth via interaction with contactin-2 and NrCAM, respectively, and in the development of post-synaptic structures via interaction with gephyrin (17).

### IgG4

**Figure 2:**
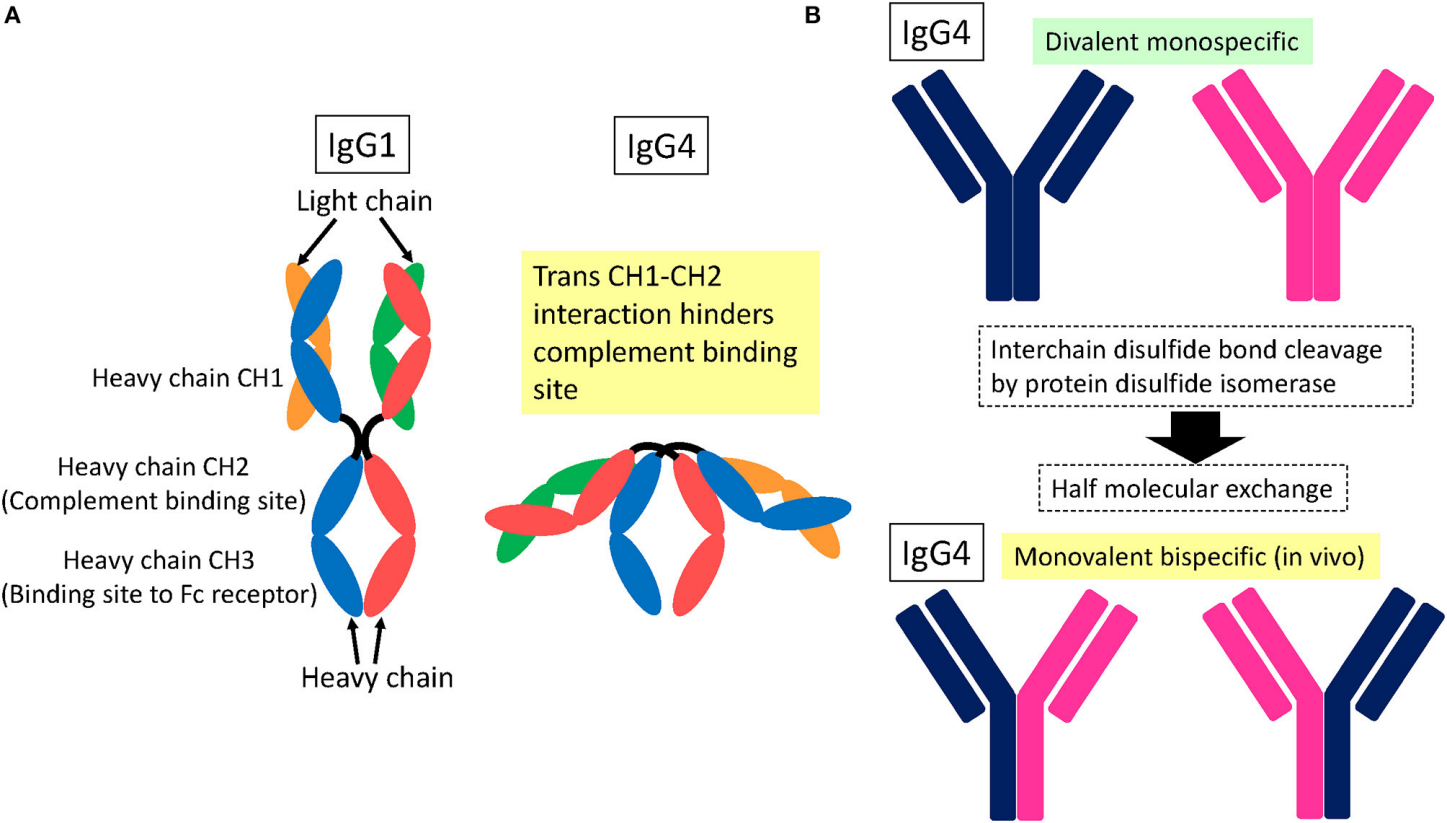
Schematic structure of IgG4. **(A)** *Trans* heavy-chain CH1–CH2 domain interaction hinders the CH2 domain within a compact structure (12). As a result, complement proteins cannot access the CH2 domain for fixation. **(B)** IgG4 is subjected to interchain disulfide bond cleavage by protein disulfide isomerase expressed on immunocytes and endothelial cells, which leads to half-molecule exchange (12). Consequently, *in vivo* IgG4 exists in a monovalent and bispecific form, which prevents IgG4 from internalizing target surface antigens.

IgG4 subclass anti-NF155 antibodies predominate in NF155^+^ CIDP (2, 4, 5). It is essential to understand the structure and function of IgG4 to elucidate the mechanism of IgG4 autoantibody-mediated nodopathy/paranodopathy. IgG4 has a compact structure arising from a *trans* heavy-chain CH1–CH2 domain interaction, which makes the CH2 domain inaccessible for complement fixation [Figure 2A; (12)]. Therefore, IgG4 cannot activate the complement cascade because it is unable to bind C1q. In addition, IgG4 exists *in vivo* in a bispecific form that is monovalent to its target. This is because of the half-molecule exchange following interchain disulfide bond cleavage by the protein disulfide isomerase expressed on immunocytes and endothelial cells [Figure 2B; (12)]. Consequently, IgG4 cannot internalize target antigens. In physiological conditions, IgG4 produced by chronic antigenic stimulation blocks allergen-specific IgE binding to allergens, thereby mitigating allergic inflammation (12). Therefore, IgG4 autoantibodies can merely block protein–protein interactions without causing full-blown inflammation.

## NF155^+^ CIDP

### Prevalence of Anti-nodal/Paranodal Protein Autoantibodies in CIDP

Initial measurements of CIDP autoantibodies against NF155 using enzyme-linked immunosorbent assays (ELISAs) revealed low positivity rates for human NF155 of 2.5% (2) and 3.8% (4), while a 22% positivity for rat NF155 was reported (26). Subsequently, a more specific antibody assay using human NF155 and flow cytometry showed positivity rates for anti-NF155 antibodies in Japanese patients with CIDP, multiple sclerosis (MS), other neuropathies, and healthy control subjects of 18% (9/50), 0% (0/32), 2.5% (1/40), and 0% (0/30), respectively (5). Sera from NF155^+^ CIDP patients bound specifically to the paranodal regions of peripheral nerves, which indicates that paranodes are the primary targets of the autoantibodies. The sera did not react with neuronal isoform NF186 (5), which indicates that the antigenic epitopes are located around the extracellular FN3 domain unique to NF155 (Figure 1B). Recently, a relatively high frequency of anti-NF155 antibody positivity (21%) was also reported for Chinese patients (27). Conversely, the latest large-scale study in European CIDP patients showed the prevalence of anti-NF155 antibodies to be 1% (15/1,500) by flow cytometry (28) and 3% (10/342) by a cell-based assay and ELISA (29). These findings indicate that anti-NF155 antibodies are more prevalent in CIDP in East-Asian populations than in European populations.

In our study of CIDP cases, all had predominantly IgG4 subclass anti-NF155 antibodies, while one anti-NF155 antibody-positive case with Guillain–Barré syndrome (GBS) had IgG1 subclass (5). Another study also reported that, in acute-onset neuropathies, including GBS, the anti-paranodal antibody subclass was IgG2/3 but not IgG4, which suggests that an IgG class switch from IgG2/3 to IgG4 may correlate with a progressive course following GBS-like onset (30). It seems likely that IgG4 subclass anti-NF155 antibodies are specific for a subset of CIDP patients.

As for other nodal/paranodal antibodies, anti-CNTN1 antibodies were detected in 6% of CIDP patients in whom advanced age, predominant motor involvement, aggressive symptom onset, and early axonal involvement were commonly observed (8–10). A recent study of anti-CNTN1 antibodies in European patients showed a prevalence of 0.7% (10/1,500) (28). Only low positivity rates (0–2%) were reported for the presence of IgG anti-NF186 antibodies in CIDP (2, 5, 28, 31). A case of CIDP with anti-CASPR1 antibodies presenting with painful neuropathy was reported (11), and the above-mentioned European study found the prevalence of anti-CASPR1 antibodies to be 0.2% (2/1,500) (28).

In summary, the prevalence of antibodies against nodal/paranodal proteins, such as NF155, CNTN1, and CASPR1, in CIDP is very low, except for the relatively high percentages of Japanese and Chinese CIDP patients who have anti-NF155 antibodies (5, 27).

### Clinical Features of IgG4 NF155^+^ CIDP

According to European (2, 4, 6) and Asian studies (5, 27), NF155^+^ CIDP patients have young onset ages and high frequencies of tremors, distal-dominant sensory and motor impairments, ataxia, and gait disturbance irrespective of ethnicity. In a Japanese cohort, IgG4 NF155^+^ CIDP mostly showed a chronic progressive course after onset (92.3%), with no patients showing acute onset (5). However, in other reports, rapidly progressive onset was observed in one in four (4) and subacute onset in 38% (12/38) of NF155^+^ CIDP patients (6). IgG4 NF155^+^ CIDP presented the following clinical features that were significantly different compared with anti-NF155 antibody-negative (NF155^−^) CIDP (5): younger age at onset [average around 25 years old (range = 13–50) vs. 48 years old (range = 13–76)], higher frequencies of drop foot (69.2 vs. 31.7%), tremor (53.8 vs. 19.5%), and gait disturbance (100 vs. 73.2%). The distal acquired demyelinating symmetric (DADS) neuropathy phenotype was significantly more frequent in NF155^+^ than in NF155^−^ CIDP (46.2 vs. 4.9%); however, the clinical subtype of NF155^+^ CIDP was not only confined to the DADS phenotype but was also associated with proximal nerve involvement presenting as the typical CIDP phenotype. Devaux et al. (6) also compared the clinical features of 38 NF155^+^ CIDP patients with those of 100 NF155^−^ CIDP patients and found sensory ataxia in 74%, tremor in 42%, and cerebellar ataxia associated with nystagmus in 13%. Overt cranial nerve manifestation is only occasionally seen in NF155^+^ CIDP, although its frequency seems to be somewhat higher in NF155^+^ CIDP than in NF155^−^ CIDP (5, 32): visual disturbance [23.1% (3/13) vs. 7.3% (3/41)], facial sensory disturbance [23.1% (3/13) vs. 17.1% (7/41)], and facial palsy [15.4% (2/13) vs. 4.9% (2/41)] (5).

### Electrophysiological Abnormalities of IgG4 NF155^+^ CIDP

#### Peripheral Nervous System

NF155^+^ CIDP usually meets the European Federation of Neurological Societies/Peripheral Nerve Society (EFNS/PNS) electrodiagnostic criteria for definite CIDP. In particular, NF155^+^ CIDP showed more pronounced prolongation of distal (7.7 ± 1.4 vs. 6.7 ± 3.3 ms) and F-wave (53.7 ± 16.3 vs. 42.4 ± 11.4 ms) latencies in the median nerve compared with NF155^−^ CIDP (5). In NF155^+^ CIDP, the distal and F-wave latencies are affected more severely than the motor conduction velocities and compound muscle action potential amplitudes (5). Together with a high frequency of spinal root hypertrophy on MRI, as described in the following section (5, 32), these findings suggest that, although all nerve segments are affected in NF155^+^ CIDP, the distal and proximal root segments tend to be involved more severely in NF155^+^ CIDP than in NF155^−^ CIDP. This may reflect the possibility that anti-NF155 antibodies have easy access to PNS tissue at nerve terminals and spinal roots where the blood–nerve barrier (BNB) is anatomically absent or loose.

Interestingly, all the NF155^+^ CIDP patients we examined showed blink reflex abnormalities, including absent and/or delayed R1 in 91.7% (11/12) and absent and/or delayed R2 in 83.3% (10/12) (32). Given the infrequent overt facial or trigeminal manifestations, a very high frequency of prolonged R1 and R2 latencies in the blink reflex test indicates that subclinical demyelination of these nerves is a common feature of NF155^+^ CIDP. Blink reflex abnormalities in CIDP have been reported to occur in 53.3% (8/15) (33) and 62.1% (36/58) (34) of Caucasians and in 90% (18/20) of Japanese (35), although anti-NF155 antibodies were not examined in these studies. Given that the prevalence of anti-NF155 antibodies is lower in Western countries (from 1 to 10% positivity) (28, 29, 36) than in Asian countries (18 and 21% positivity) (5, 27), it is conceivable that these results mostly reflected NF155^−^ CIDP cases, particularly for Caucasians. Because all the NF155^+^ CIDP patients we examined had abnormal blink reflex, a higher frequency of blink reflex abnormalities is indicated for NF155^+^ CIDP compared with NF155^−^ CIDP. The higher frequency of NF155^+^ CIDP in Asians (5, 27) than in Caucasians (28, 29, 36) may be responsible in part for the relatively higher frequency of blink reflex abnormalities in Japanese CIDP patients (35) compared with Caucasian CIDP patients (33, 34).

**Figure 3:**
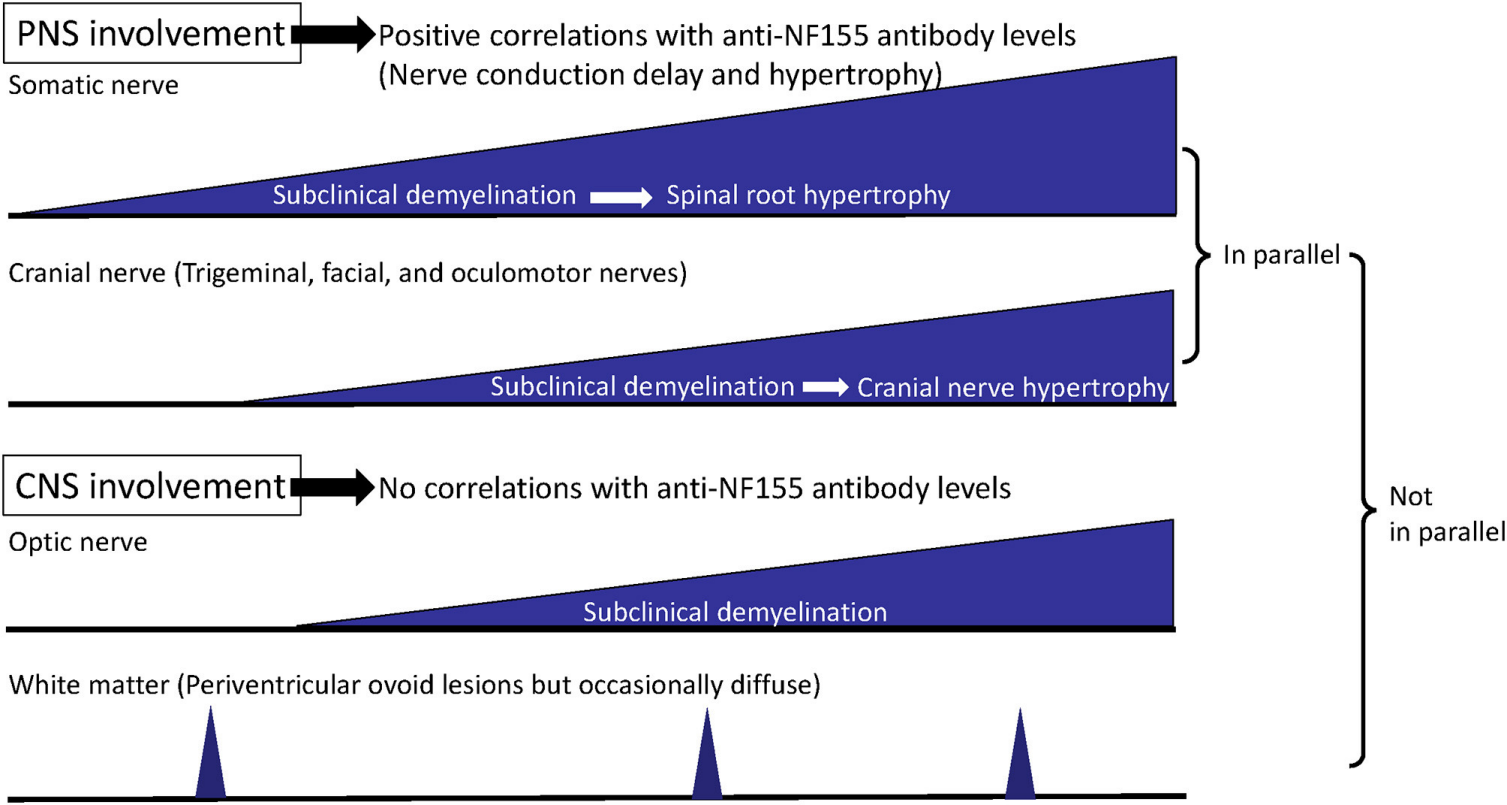
Process of disease progression in NF155^+^ chronic inflammatory demyelinating polyneuropathy (CIDP)/combined central and peripheral demyelination (CCPD). In NF155^+^ CIDP/CCPD, the disease initiates (subclinical) demyelination at the distal nerve terminals of the somatic nerve and then extends to the cranial nerves. Later, hypertrophy of the proximal spinal nerve and roots occurs over the long disease duration, which is followed by hypertrophy of the cranial nerves, such as trigeminal and oculomotor nerves. Somatic and cranial nerve involvements develop in parallel except for the optic nerve, while peripheral nervous system (PNS) and central nervous system (CNS) involvements do not occur in parallel.

In our study, the R1 latencies on the stimulation of either side had significant positive correlations with the anti-NF155 antibody levels (right: *r* = 0.9184; left; *r* = 0.9217) (32), which strongly supports a pathogenic role of anti-NF155 antibodies in trigeminal and facial nerve involvement. In addition, the R1 latencies had strong positive correlations with the distal and F-wave latencies of the median and ulnar nerves (32), which indicates that somatic and cranial nerves are involved in parallel via anti-NF155 antibodies (Figure 3). The fact that the distal and F-wave latencies correlated with the blink reflex abnormalities more strongly than with the motor conduction velocities and compound muscle action potential amplitudes probably indicates an antibody-mediated nerve terminal damage in the trigeminal and facial nerves, as described above for somatic nerves (32).

#### Central Nervous System

In our NF155^+^ CIDP patient cohort, absent and/or prolonged visual-evoked potentials (VEPs) were observed in 10 of 13 (76.9%) patients and in 17 of 26 (65.4%) eyes (32), although clinically overt visual disturbance was infrequent. Otherwise, VEPs in CIDP were examined only in a few studies on a small series of Caucasian patients without investigating anti-NF1555 antibodies (37–39). In these studies, 47% (8/17) (37), 50% (9/18) (38), and 50% (5/10) of patients (39) showed abnormalities on VEPs. It appears that NF155^+^ CIDP patients have a higher frequency of abnormal VEPs (76.9%) compared with the abnormal VEP frequencies reported in the total CIDP patients (47–50%). The latter mostly reflects NF155^−^ CIDP cases because the frequency of NF155^+^ CIDP was very low in Caucasians (28, 29, 36). As VEPs were evoked by monocular full visual field stimulation, markedly prolonged P100 latencies in NF155^+^ CIDP patients without retinal and occipital lesions suggest that subclinical demyelination exists in the optic nerves. Because NF155 is present in the terminal loop of oligodendrocytes as well as Schwan cells (36), it is possible that anti-NF155 antibodies frequently afflict the optic nerves. This is in accord with the occasional development of CNS white matter lesions suggestive of demyelination in patients with NF155^+^ CCPD and CIDP (3, 40). Collectively, anti-NF155 antibodies are regarded to be associated with CIDP and CCPD phenotypes.

### Neuroimaging Abnormalities of IgG4 NF155^+^ CIDP

#### Peripheral Nervous System

By three-dimensional nerve-sheath signal increased by inked rest-tissue rapid acquisition with relaxation enhancement imaging (3D SHINKEI), a new MRI neurography method to visualize spinal roots and plexuses (41), all 13 patients with IgG4 anti-NF155 antibodies studied commonly demonstrated remarkable hypertrophy of the cervical and lumbar spinal roots (5, 32). In the largest diameters of the bilateral C5–C8 roots, NF155^+^ CIDP patients had significantly greater values than did NF155^−^ CIDP patients (7.7 ± 1.3 vs. 4.9 ± 2.0 mm) (5, 32, 41). The root diameters tended to have a positive correlation with disease duration in patients with NF155^+^ CIDP (*r* = 0.739) (5). Hypertrophy of the proximal oculomotor and trigeminal nerves, which was termed the Mustache sign, was also reported in a case of NF155^+^ CIDP (42). In our Japanese cohort, hypertrophy and high signal intensity of the trigeminal nerves were found in 69.2% (9/13) and 76.9% (10/13), respectively (32). The intra-orbital trigeminal nerve width on coronal sections positively correlated with disease duration (right side: *r* = 0.7835; left side: *r* = 0.7857) (32), as shown in the somatic nerves. Accordingly, trigeminal nerve hypertrophy affecting all three branches is a common feature of patients with NF155^+^ CIDP who have a longstanding clinical course. The cranial nerve hypertrophy may be a useful marker for NF155^+^ CIDP. The nerve hypertrophy gradually proceeds over a long disease course, given the significant positive correlation between nerve width and disease duration in both somatic and cranial nerves. However, subclinical demyelination appears to precede nerve hypertrophy because the frequency of R1 abnormality in the blink reflex test was much higher than that of trigeminal nerve hypertrophy (Figure 3). Furthermore, all 13 NF155^+^ CIDP patients studied showed hypertrophy of the cervical and/or lumbosacral nerve roots, whereas 10 of the 13 (69.2%) patients had trigeminal nerve hypertrophy (32), which suggests that somatic nerve hypertrophy precedes cranial nerve hypertrophy (Figure 3).

#### Central Nervous System

IgG4 NF155^+^ CIDP patients also occasionally develop white matter lesions, suggestive of demyelination in the CNS, which is designated CCPD (3, 5, 6, 16). CNS demyelinating lesions on MRI were reported in 33.3% (3/9) (5) and 8% (3/38) of NF155^+^ CIDP patients (6). However, we detected no morphological or signal abnormalities in the optic nerves of any of our NF155^+^ CIDP patients, including those with VEP abnormalities (32). The trigeminal nerve exit zones where oligodendrocytes exist did not show any hypertrophy (32). Therefore, nerve hypertrophy only develops in the cranial nerves insulated by PNS myelin and Schwann cells, but not by CNS myelin and oligodendrocytes (Figure 3).

### Neuropathology of NF155^+^ CIDP

There have been no autopsy reports for NF155^+^ CIDP. However, histological examinations of the biopsied sural nerves showed subperineurial edema and occasional paranodal demyelination, but no vasculitis, inflammatory cell infiltrates, or onion bulbs (5). Surprisingly, loss of myelinated fibers was mild even years after disease onset (5). In the biopsied skin specimens, elongation of the nodes of Ranvier, as determined by CASPR1 immunostaining, with loss of NF155 immunoreactivity was also reported in NF155^+^ CIDP (29). Electron microscopy studies revealed the detachment of terminal Schwann cell loops from axons at paranodes with disruption of septate-like transverse bands in NF155^+^ CIDP cases, but not in NF155^−^ CIDP cases (43–45). In CIDP, macrophages phagocytose myelin, initiating at either nodal regions or internodes (46). However, such a macrophage-induced demyelination was not observed in NF155^+^ CIDP (46). Similar elongation of the nodes of Ranvier in dermal myelinated fibers (9, 29) and disruption of septate-like transverse bands accompanied with the detachment of Schwann cell terminal loops from axons at the paranodes were also detected in anti-CNTN1 antibody-positive CIDP (43). Therefore, it is plausible that anti-NF155 antibodies disrupt NF155 interaction with the CNTN1/CASPR1 complex without eliciting a severe inflammatory response at paranodes, which leads to the detachment of Schwann cell terminal loops from the axons and to conduction failure.

### Cerebrospinal Fluid Abnormalities of NF155^+^ CIDP

NF155^+^ CIDP commonly shows extremely high CSF protein levels. In our study, the CSF protein levels were significantly higher in NF155^+^ CIDP compared with NF155^−^ CIDP patients (317.0 ± 141.1 vs. 103.8 ± 75.8 mg/dl) (5, 13). Even the CSF cell counts showed a small but significant increase in NF155^+^ CIDP (4.0 ± 3.1) compared with NF155^−^ CIDP (2.1 ± 2.3) and other non-inflammatory neurological disease (NIND) patients (1.8 ± 1.9) (13). These observations indicate severe spinal root inflammation in NF155^+^ CIDP, although the biopsied sural nerve specimens had no inflammatory cell infiltrate.

We measured 28 CSF cytokines, chemokines, and growth factors by a multiplexed fluorescence immunoassay in a relatively large cohort of NF155^+^ CIDP patients (*n* = 35) and compared them with those of NF155^−^ CIDP (*n* = 36) and NIND patients (*n* = 28) (13). In NF155^+^ CIDP, the levels of CXCL8/interleukin 8 (IL8), IL13, tumor necrosis factor alpha (TNFα), CCL11/eotaxin, CCL2/MCP1, and IFNγ were significantly higher and the levels of IL1β, IL1ra, and GCSF were significantly lower compared to those in NIND. Compared with NF155^−^ CIDP, the levels of CXCL8/IL8 and IL13 were significantly higher and the levels of IL1β, IL1ra, and IL6 were significantly lower in NF155^+^ CIDP. Importantly, the CXCL8/IL8, IL13, CCL11/eotaxin, CXCL10/IP10, CCL3/MIP1α, CCL4/MIP1β, and TNFα levels were positively correlated with the markedly elevated CSF protein levels, and the IL13, CCL11/eotaxin, and IL17 levels were positively correlated with the increased CSF cell counts. Conversely, NF155^−^ CIDP had significantly increased IFNγ levels compared with NIND and exhibited positive correlations of the IFNγ, CXCL10/IP10, and CXCL8/IL8 levels with the CSF protein levels. According to the canonical discriminant analysis of cytokines/chemokines, NF155^+^ and NF155^−^ CIDP were separable, with IL4, IL10, and IL13 the three most significant discriminators (13). These three cytokines are all required for class switching to IgG4. In NF155^+^ CIDP, both Th2 and Th1 cytokines are likely to be involved, whereas only Th1 cytokines appear to be involved in NF155^−^ CIDP. In particular, Th2 cytokines appear to be critical in inducing intrathecal inflammation, as shown by the positive correlations with the increased CSF protein levels and cell counts. IL13, CXCL8/IL8, CCL4/MIP1β, CCL3/MIP1α, and CCL5/RANTES were decreased by combined immunotherapies in nine NF155^+^ CIDP patients in parallel with clinical improvement (13), which further supports the critical roles of Th2 cytokines in this condition.

**Figure 4:**
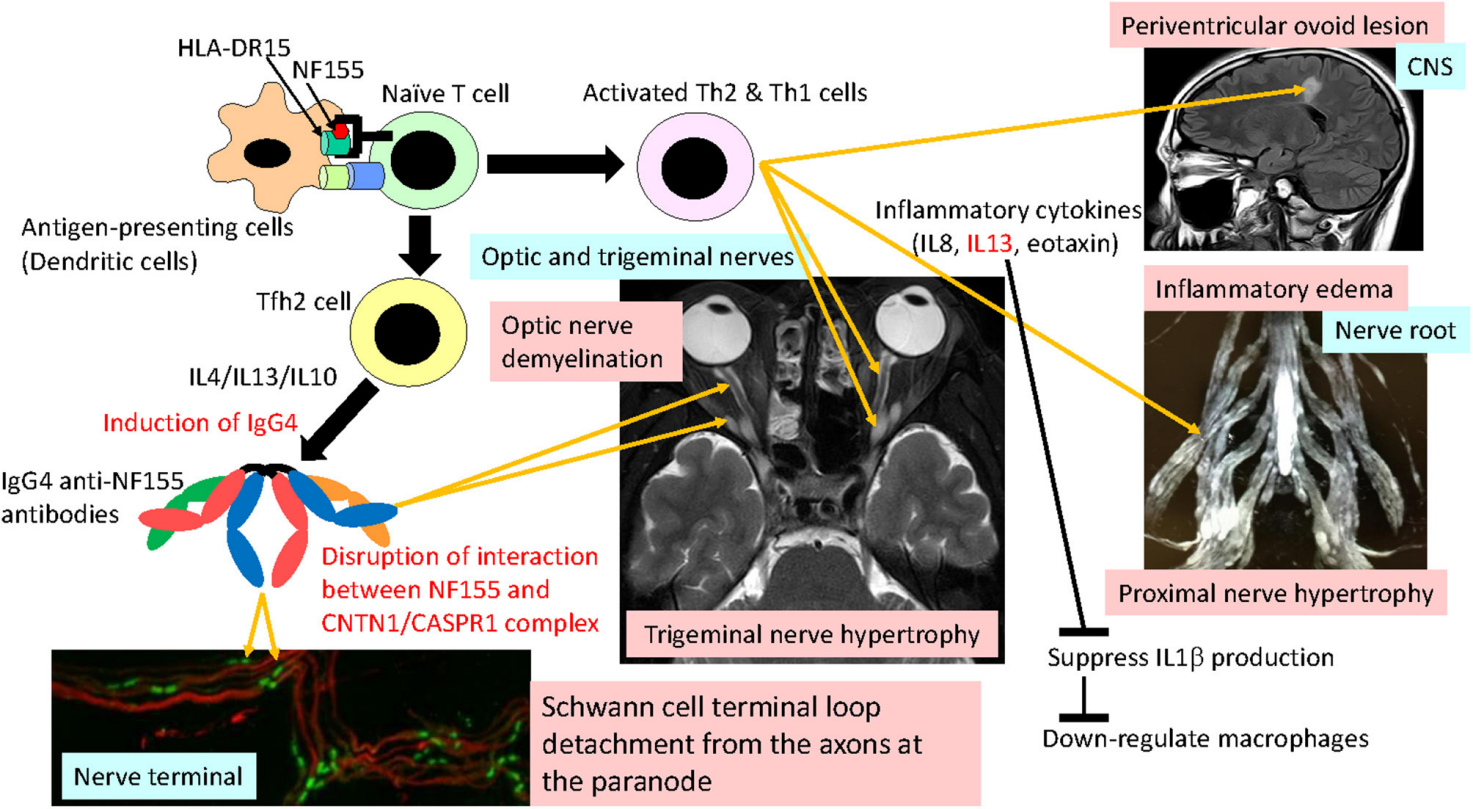
Hypothetical mechanism of NF155^+^ chronic inflammatory demyelinating polyneuropathy (CIDP)/combined central and peripheral demyelination (CCPD). NF155 peptides are presented by a DRB1*15:01/DRB1*15:02 and/or DQA1*01:02-DQB1*06:02/DQA1*01:03-DQB1*06:01 complex to naive T cells, initiating Tfh2/Th1 cell differentiation. Tfh2 cells produce IL4/IL13/IL10, which induce IgG4 class switching. IgG4 anti-NF155 antibodies invade the nerve terminal and nerve roots where the blood nerve barrier is absent or leaky. Invaded anti-NF155 antibodies disrupt the interaction between NF155 and the CNTN-1/CASPR1 complex at the paranode, which leads to Schwann cell terminal loop detachment from axons. Activated Th2 and Th1 cells cause inflammation at the spinal roots, resulting in nerve root hypertrophy, occasionally producing periventricular ovoid lesions in the central nervous system (CNS). Overproduction of IL13 downregulates IL1β production, which depresses macrophage activation and recruitment. Cranial nerves, such as trigeminal, facial, oculomotor, and optic nerves, are also affected by anti-NF155 antibodies and possibly by activated Th2/Th1 cells.

Interestingly, in NF155^+^ CIDP, a significantly depressed IL1β was recovered to the normal range after immunotherapy (13), which suggests that the decrease in IL1β was not coincidental but associated with the disease process. The downregulation of IL1β is consistent with the absence of macrophage-mediated demyelination in this condition (46). Th2 cytokines, such as IL13, reportedly downregulate IL1β. Thus, it is possible that overrepresented Th2 cytokines play critical roles in inducing IgG4 autoantibodies via the effects of IL4/IL13/IL10 and spinal root inflammation on the one hand and by downregulating IL1β and macrophage functions on the other (Figure 4).

### Genetic Risk for IgG4 NF155^+^ CIDP

Certain human leukocyte antigen (HLA) class II alleles are strongly associated with IgG4 autoantibody-mediated diseases: HLA-DRB1^*^07:01-DQB1^*^02:02 with anti-leucine-rich, glioma-inactivated 1 antibody-positive autoimmune encephalitis (47), *HLA-DR14-* and *HLA-DR16-DQ5* haplotypes with anti-muscle-specific kinase (MuSK) antibody-positive myasthenia gravis (48, 49), HLA-DRB1^*^15:01 and HLA-DRB3^*^02:02 with membranous nephropathy with autoantibodies to phospholipase A2 receptor (50), and HLA-DRB1^*^11 in thrombotic thrombocytopenic purpura with autoantibodies to a disintegrin and metalloproteinase with a thrombospondin type 1 motif, member 13 (51). It was recently reported that the frequency of the HLA-DRB1^*^15 allele was significantly higher in 13 NF155^+^ CIDP patients from various European countries (Spain, France, Italy, and UK; 92% Caucasians) than in the general Spanish population (52). Surprisingly, all 22 Japanese patients with NF155^+^ CIDP had either of two specific *HLA* haplotypes: HLA-DRB1^*^15:01-DRB5^*^01:01-DQA1^*^01:02-DQB1^*^06:02 or HLA-(A^*^24:02)-B^*^52:01-C^*^12:02-DRB1^*^15:02-DRB5^*^01:02-DQA1^*^01:03-DQB1^*^06:01. Consequently, the HLA-DRB1^*^15, HLA-DRB1^*^15:01, HLA-DQB1^*^06:01/06:02, HLA-DQB1^*^06:02, and HLA-DRB1^*^15:01-DQB1^*^06:02 frequencies were significantly greater in NF155^+^ CIDP patients than in healthy Japanese controls (53). These findings indicate an involvement of specific HLA class II molecules in the pathogenesis of IgG4 NF155^+^ CIDP. Given that clinical features are similar between the two haplotype groups in our study, common or similar NF155 peptides are presented by these HLA class II molecules to T cells in both *HLA* haplotypes. The higher frequency of IgG4 NF155^+^ CIDP in the Japanese compared with Caucasian populations (5, 28, 29) may partly be explained by the fact that this *HLA* haplotype containing HLA-DRB1^*^15:02 is overexpressed in the general Japanese population, whereas HLA-DRB1^*^15:02 is rare in Caucasians (54). DQA1^*^01:02 and DQA1^*^01:03, and DQB1^*^06:01 and DQB1^*^06:02, have five and 26 amino acid differences, respectively (55), whereas DRB1^*^15:01 and DRB1^*^15:02 have only one amino acid difference at the 86th amino acid, which is located in pocket 1 of the peptide binding groove (56, 57). The shared core sequences of NF155, which strongly bind to not only DRB1^*^15:01 and DQA1^*^01:02-DQB1^*^06:02 but also to DRB1^*^15:02 and DQA1^*^01:03-DQB1^*^06:01, were predicted by *in silico* analysis (53). Of note is that the two core sequences (amino acids 336–344 and 845–853 of NF155) that are shared between DRB1^*^15:01 and DRB1^*^15:02 substantially overlap with the two core sequences (amino acids 339–347 and 848–856) shared by DQA1^*^01:03-DQB1^*^06:01 and DQA1^*^01:02-DQB1^*^06:02 (53). Therefore, it is possible that these peptides shared by all four heterodimers are presented by not only DRB1^*^15:01 and DRB1^*^15:02 but also by DQA1^*^01:03-DQB1^*^06:01 and DQA1^*^01:02-DQB1^*^06:02. Among these shared peptide sequences, the one with the strongest affinity (amino acids 336–344) may be a good candidate for a T cell epitope on NF155.

### NF155^+^ CCPD

#### General Features of CCPD

CCPD is an extremely rare disease involving both CNS and PNS. According to the nationwide survey of CCPD in Japan (16), CCPD probably affects <0.52% of MS and 2.8% of CIDP patients. The male/female ratio was 1:2.6. The age at onset was 32 years, on average, although it was widely varied from 8 to 59 years. The onset mode was acute in 19.4%, subacute in 45.2%, and chronic in 35.5% of patients. As for clinical courses, 26.3% of patients were monophasic, 52.6% were relapsing–remitting, and 21.1% were chronic progressive. CCPD patients with simultaneous onset of CNS and PNS involvement showed greater disability and more frequent extensive cerebral and spinal cord MRI lesions, but less recurrence than those with temporarily separated onset, while optic nerve involvement was more frequent in the latter patients (16). Severe disability (Hughes functional scale score ≥4), with some requiring artificial ventilation, was seen in 40.0% of CCPD patients at the peak of illness, while 65.0% had no or only mild disability (Hughes functional scale score ≤1) after immunotherapy (16). Intravenous or oral corticosteroids were most frequently used in CCPD patients, followed by IVIg, which resulted in improvements in 83.3, 75.0, and 66.7% of patients, respectively (16). A small fraction of CCPD patients received plasmapheresis, which led to improvements in 87.5%. However, interferon-β was efficacious only in 10.0% and even exacerbated the symptoms in 30.0% of patients (16).

#### Anti-NF155 Antibodies in CCPD

Kawamura et al. (3) first discovered anti-NF155 antibodies in CCPD cases. Anti-NF155 antibodies are present in 45% of CCPD cases according to a nationwide survey in Japan (16). However, the positivity rates for anti-NF155 antibodies in CCPD differ among studies and ethnicities, possibly reflecting the different assay methods and antigen species used. NF155^+^ CCPD patients demonstrate: (i) sequential or simultaneous CNS and PNS involvement; (ii) diffuse conduction slowing with focal conduction failure indistinguishable from that in CIDP in nerve conduction studies; (iii) CNS involvement mostly typical for MS, in which spinal cord lesions and gadolinium-enhanced lesions could develop, but occasionally atypical involvement, with diffuse cerebral white matter lesions; (iv) negative CSF oligoclonal IgG bands; and (v) beneficial effects of combined immunotherapies including corticosteroids, IVIg, and plasma exchange on both CNS and PNS lesions (3, 16). As stated above, a high frequency of optic nerve involvement is characteristic of NF155^+^ CIDP (32). However, we found no correlations between VEP abnormalities and the somatic nerve conduction study (NCS) parameters (32), which suggests that PNS and CNS involvement may not develop in parallel (Figure 3) and that some distinct mechanisms may underlie the differences between PNS and CNS damage. In summary, NF155^+^ CIDP characteristically shows frequent subclinical CNS involvement suggestive of demyelination, while overt clinical CNS manifestations are infrequent. NF155 is one of the antigens responsible for CCPD, but other relevant antigens warrant investigation.

### Treatment Response in NF155^+^ CIDP

NF155^+^ CIDP is refractory to IVIg (4, 5), which is usually effective in ~60% of all CIDP patients (58, 59). Devaux et al. (6) reported that 80% (20/25) of NF155^+^ CIDP patients had poor response to IVIg. The plausible mechanisms of action for IVIg in CIDP include the inhibition of the complement pathway, modulation of Fc receptors on macrophages, anti-idiotype antibody production, inhibition of cell migration by the modulation of adhesion molecules, and promotion of remyelination (60). Although the exact mechanism of NF155^−^ CIDP remains to be elucidated, one study reported that, in some NF155^−^ CIDP patients, macrophages strip myelin via the Fc receptor and/or complement-mediated mechanisms (antibody- or complement-dependent phagocytosis) (46). Accordingly, it is possible that IVIg is beneficial for NF155^−^ CIDP partly by inhibiting complement activation and modulating Fc receptors on macrophages. The poor response to IVIg in NF155^+^ CIDP may be explained by the lack of complement-mediated inflammatory cascade and macrophage-mediated demyelination, as shown by pathological studies of biopsied sural nerves (5, 44, 46).

Instead, a retrospective evaluation showed that corticosteroids combined with IVIg were more beneficial than IVIg alone (5), which is compatible with the widespread use of corticosteroids in the treatment of diseases related to disease-specific IgG4 autoantibodies, such as pemphigus (61) and thrombotic thrombocytopenic purpura (62). In NF155^+^ CIDP, long-term immunosuppression by daily oral corticosteroids and/or immunosuppressants is more efficacious for sustained improvement than repeated IVIg administrations. Notably, in a long-term observational study, the anti-NF155 antibody levels varied in parallel with clinical and electrophysiological changes, or even preceded these changes (63). These observations further support a pathogenic role of IgG4 anti-NF155 antibodies themselves. Recently, rituximab, an anti-CD20 monoclonal antibody targeting B cells, was tried in a small series of patients with IgG4 NF155^+^ CIDP who were refractory to conventional immunotherapies and was found to be of substantial benefit [effective in two of three cases in Querol et al. (64); two of three in Roux et al. (65); one of one in Cortese et al. (29); two of two in Stengel et al. (66); one of two in Godil et al. (67); and in all eight cases in Rasband and Peles (25)] along with decreased anti-NF155 antibody titers in some (64).

### Mechanism of IgG4 NF155^+^ CIDP

IgG4 autoantibodies merely block protein–protein interactions without activating the complement cascade or internalizing target antigens. Therefore, the primary effect of IgG4 anti-NF155 antibodies is likely to be the blockade of interactions between NF155 and CNTN1/CASPR1, which leads to Schwann cell terminal loop detachment from axons at paranodes, as seen in the biopsied sural nerve pathology (5, 44, 46). The restoration of nerve conduction by plasma exchange associated with decreasing anti-NF155 antibody titers is also compatible with IgG4 acting as blocking antibodies (63). Passive transfer experiments revealed that IgG4 antibodies targeted NF155 on Schwann cell surfaces, diminished the NF155 protein levels, and prevented the new formation of paranodal complexes (68). In the case of anti-CNTN1 antibodies, intraneurally injected IgG4 accessed the paranode borders near the nodal lumen and filled the paranodal segments, which led to loss of the paranodal complex, whereas IgG1 did not pass the paranodal barrier (68). This may relate to the compact structure of IgG4 (12), which makes it easy for IgG4 autoantibodies to approach the paranodal space. However, it was shown that IgG4 anti-NF155 antibodies did not penetrate the paranodal regions like IgG4 anti-CNTN1 antibodies did (68). Passive transfer experiments revealed that IgG4 antibodies targeted NF155 on Schwann cell surfaces, diminished the NF155 protein levels, and prevented the new formation of paranodal complexes in mature nervous tissues (69). Collectively, it is assumed that IgG4 anti-NF155 antibodies invade the PNS tissue at nerve terminals where the BNB is anatomically absent and cause Schwann cell terminal loop detachment via perturbation of new NF155-CNTN1/CAPR1 complex formation at paranodes (Figure 4).

Conversely, the extensive proximal nerve hypertrophy and the pronounced CSF protein elevation, suggesting severe inflammation, and/or edema of nerve roots, which is unique to this condition, are difficult to explain solely by IgG4 antibody functions. A small but significant increase in the CSF cell counts in pretreated NF155^+^ CIDP patients also supports the presence of intrathecal inflammation (13). The levels of pro-inflammatory cytokines/chemokines, such as IL13, CCL11/eotaxin, and CXCL8/IL8, were increased in NF155^+^ CIDP patients and showed significant positive correlations with the CSF protein levels (13); therefore, these cytokines/chemokines and the Th2 cells producing them may be directly or indirectly involved in nerve root inflammation and BNB destruction [Figure 4; (70, 71)].

In CCPD, periventricular ovoid lesions are occasionally seen, which are likely caused by perivenous inflammatory demyelination initiated by T cells (3). Intriguingly, the numbers of NF155-specific Th1 cells producing IFNγ were increased before clinical CNS manifestations and were assumed to cause CNS inflammation (72). The length of contact time between the HLA class II molecule–peptide complex and the T cell receptor (TCR) determines the differentiation of Th1 and T follicular helper (Tfh) cells, which play central roles in producing antibodies (73, 74). Thus, it is possible that the NF155 peptide–DRB1^*^15:01/DRB1^*^15:02 and/or NF155 peptide–DQA1^*^01:02-DQB1^*^06:02/DQA1^*^01:03-DQB1^*^06:01 complex dictates Tfh2/Th1 cell differentiation in IgG4 NF155^+^ CIDP/CCPD (Figure 4).

## Conclusions

### Diagnostic and Treatment Strategy for NF155^+^ CIDP

Awareness of IgG4 nodal antibody-positive CIPD is important in clinical practice because IgG4 autoantibody-related neurological diseases are often refractory to conventional immunotherapies, such as IVIg. In NF155^+^ CIDP patients, the measurement of anti-NF155 antibodies is recommended in those who meet the EFNS/PNS electrodiagnostic criteria and have high CSF protein levels (i.e., higher than 100 mg/dl). Hypertrophy of nerve roots and cranial nerves as well as VEP and blink reflex test abnormalities are also suggestive of NF155^+^ CIDP. Anti-NF155 antibodies should also be examined in patients with CCPD manifestations. As IVIg alone is not sufficient to treat NF155^+^ CIDP and nerve hypertrophy responds poorly to immunotherapy after long disease duration (5), early introduction of combined therapy with corticosteroids and immunosuppressants is recommended. Because rapid tapering of these drugs may cause relapse, long-term use of low-dose corticosteroids and immunosuppressants is helpful, together with monitoring of the anti-NF155 antibody levels (63).

### Future Perspectives

The mechanism by which IgG4 anti-NF155 antibodies cause peripheral nerve demyelination by Schwann cell terminal loop detachment from axons has been well-characterized clinically and experimentally, while the mechanisms of severe nerve root and cranial nerve hypertrophy, as well as frequent involvement of CNS tissues, such as optic nerves, remain to be elucidated. In particular, as NF155^+^ CIDP is strongly associated with certain *HLA* class II alleles (52, 53), further studies on HLA class II molecule-restricted T cells are warranted to clarify the mechanism for this condition.

Another important issue is to elucidate the mechanism by which IgG4 antibodies against nodal/paranodal proteins emerge. IgG4 class switching requires help from Tfh2 cells producing IL4, IL10, and IL13. Given that IgG4 antibodies act as blocking antibodies to alleviate allergic inflammation by interfering with the binding of allergen-specific IgE to allergens, environmental antigens that cross-react with nodal/paranodal proteins may be important for future investigations.

Concerning treatment, clinical trials of an anti-B cell monoclonal antibody therapy are currently being undertaken in NF155^+^ CIDP patients (75). NF155^+^ CIDP affects young adults and may cause severe disability, unless appropriate combined immunotherapy is introduced in the early course of the disease. Considering the long-term side effects of the corticosteroids and immunosuppressants currently used to suppress relapse in NF155^+^ CIDP patients, more efficacious and safer drugs for NF155^+^ CIDP treatment need to be developed.

## Author Contributions

J-iK contributed to the study conception and design, obtained funding and did the acquisition, analysis, and interpretation of data and the drafting of the manuscript.

## Conflict of Interest

J-iK received research funds from Dainippon Sumitomo Pharma, Daiichi Sankyo, Mitsubishi Tanabe Pharma, and Kyowa Kensetsukougyo, and consultancy fees, speaking fees and/or honoraria from Novartis Pharma, Mitsubishi Tanabe Pharma, CSL Behring, Biogen Japan, Teijin Health Care, the Takeda Pharmaceutical Company, Kyowa Kirin, Ono Pharmaceutical Co. Ltd., Alexion Pharmaceuticals Inc., Tsumura, Ricoh, EMC, and Eisai.
